# Supplementary material for: Metabolic and Anti-Inflammatory Protective Properties of Human Enriched Serum following Artichoke Leaf Extract Absorption: Results from an Innovative Ex Vivo Clinical Trial
Source: Nutrients. 2021 Jul 30;13(8):2653. doi: 10.3390/nu13082653 (PMC8398945; doi:10.3390/nu13082653)
Supplement: Supplementary file 1 [file nutrients-13-02653-s001.zip › nutrients-1275257-supplementary.pdf]

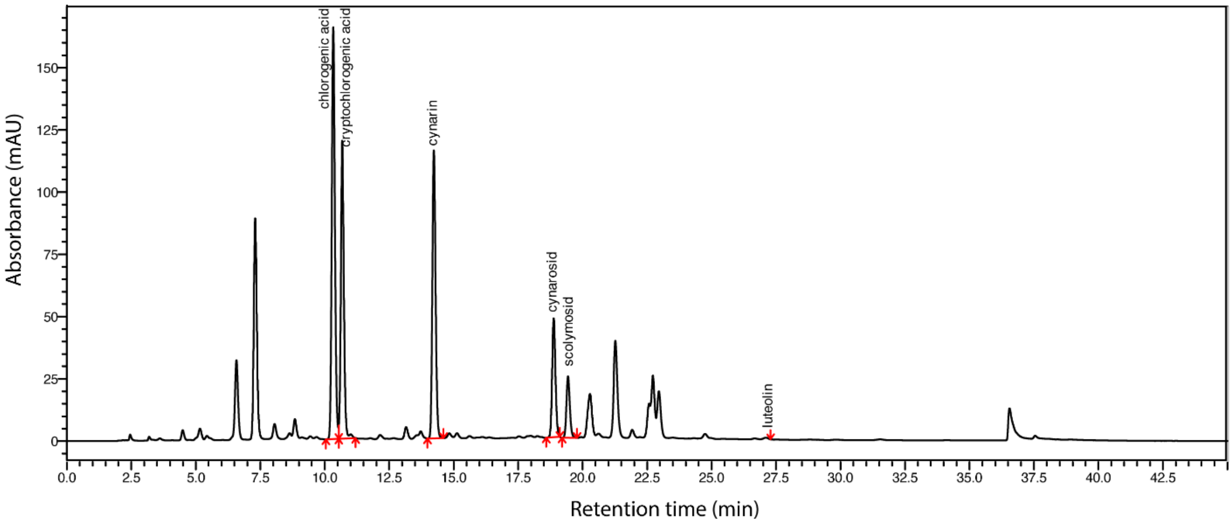

Figure S1. HPLC chromatogram of artichoke leaf extract. The chromatographic analysis was carried out according to the European Pharmacopoeia (Ph. Eur. 10.0, 2389 (04/2018). External standards purchased from Extrasynthese were used to identify chlorogenic acid, cryptochlorogenic acid, cynarin, cymaroside, scolymosid and luteolin.

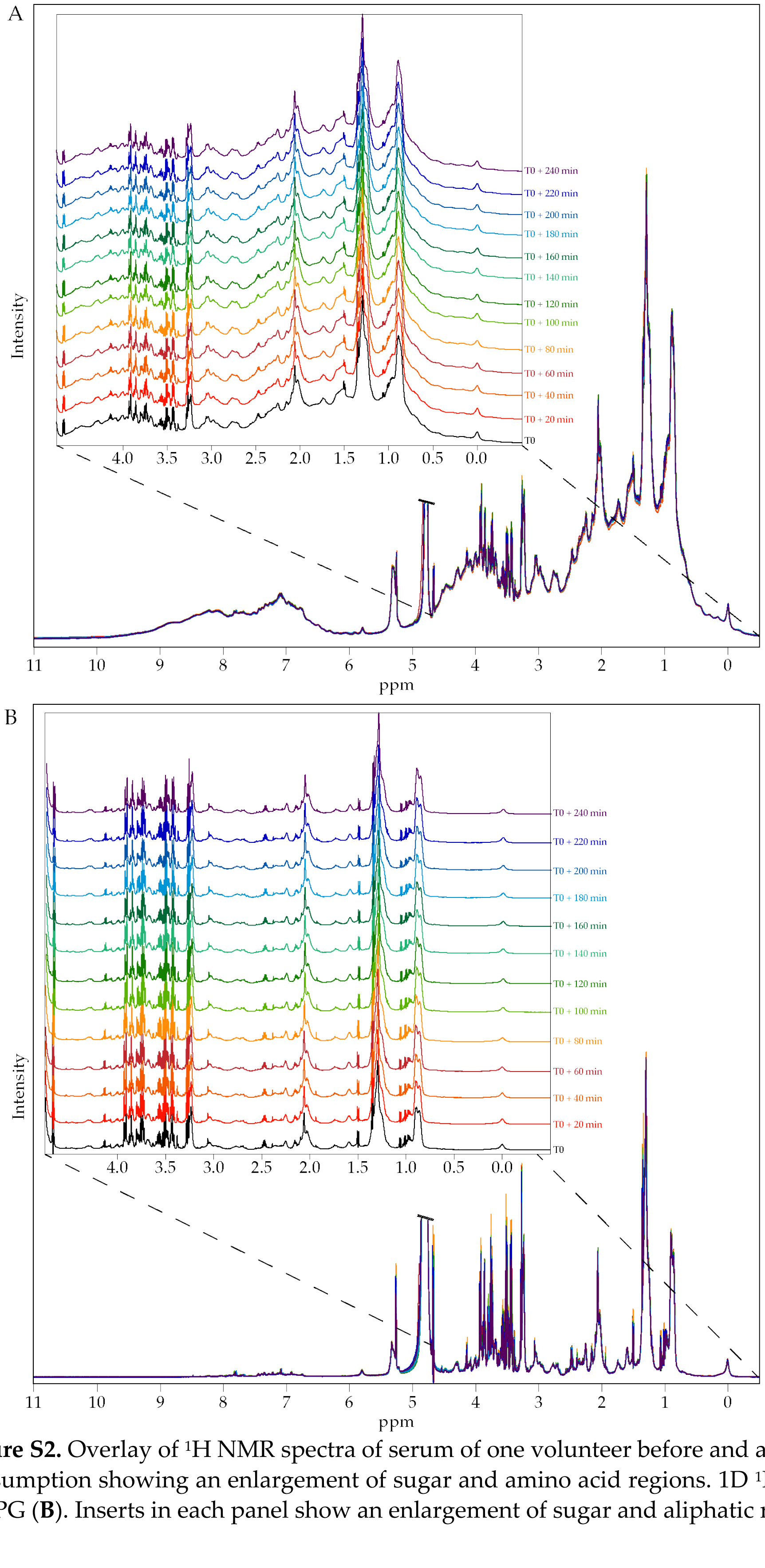

Figure S2. Overlay of ^{1}H NMR spectra of serum of one volunteer before and after artichoke leaf extract consumption showing an enlargement of sugar and amino acid regions. 1D ^{1}H NOESY (**A**) and 1D ^{1}H CMPG (**B**). Inserts in each panel show an enlargement of sugar and aliphatic regions.

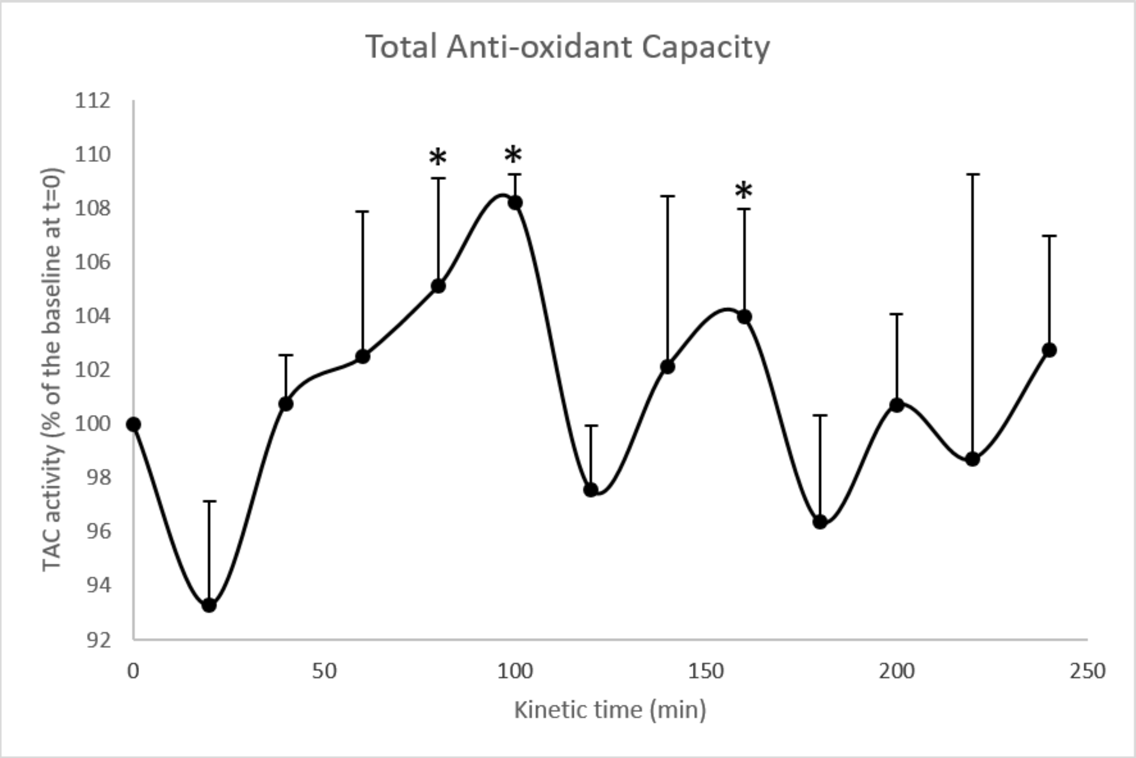

Figure S3: Total antioxidant capacity of human serum samples following artichoke leaf extract ingestion. The Total Antioxidant Capacity Assay Kit (Sigma-Aldrich MAK187) was used to determine the antioxidant capacity of serum samples according to supplier's recommendations. Briefly, Cu2+ ion is converted to Cu+ by antioxidant molecules and proteins. The use of the Protein Mask prevents Cu2+ reduction by protein, enabling the analysis of antioxidant small molecules. The reduced Cu+ ion chelates with a colorimetric probe, giving a broad absorbance peak at 570 nm, which is proportional to the total antioxidant capacity. Trolox, a water-soluble vitamin E analog, serves as an antioxidant standard. The kit gives antioxidant capacity in Trolox equivalents (ranging from 4–20 nmol/well). The 100 min time frame for maximum enrichment revealed by NMR spectroscopy was confirmed by the measurement of the total antioxidant capacity of the collected serum fractions (* $p < 0{,}05$ vs t = 20 min).
